# Sodium-calcium exchangers in rat trigeminal ganglion neurons

**DOI:** 10.1186/1744-8069-9-22

**Published:** 2013-04-29

**Authors:** Hidetaka Kuroda, Ubaidus Sobhan, Masaki Sato, Maki Tsumura, Tatsuya Ichinohe, Masakazu Tazaki, Yoshiyuki Shibukawa

**Affiliations:** 1 Oral Health Science Center hrc8, Tokyo Dental College, Tokyo 261-8502, Japan; 2 Department of Dental Anesthesiology, Tokyo Dental College, Tokyo 261-8502, Japan; 3 Department of Physiology, Tokyo Dental College, Tokyo 261-8502, Japan

**Keywords:** Calcium homeostasis, Sodium-calcium exchangers, Orofacial pain, Trigeminal neuron, Voltage-dependent Na^+^ channels

## Abstract

**Background:**

Noxious stimulation and nerve injury induce an increase in intracellular Ca^2+^ concentration ([Ca^2+^]_i_) via various receptors or ionic channels. While an increase in [Ca^2+^]_i _excites neurons, [Ca^2+^]_i_ overload elicits cytotoxicity, resulting in cell death. Intracellular Ca^2+^ is essential for many signal transduction mechanisms, and its level is precisely regulated by the Ca^2+ ^extrusion system in the plasma membrane, which includes the Na^+^-Ca^2+ ^exchanger (NCX). It has been demonstrated that Ca^2+^-ATPase is the primary mechanism for removing [Ca^2+^]_i _following excitatory activity in trigeminal ganglion (TG) neurons; however, the role of NCXs in this process has yet to be clarified. The goal of this study was to examine the expression/localization of NCXs in TG neurons and to evaluate their functional properties.

**Results:**

NCX isoforms (NCX1, NCX2, and NCX3) were expressed in primary cultured rat TG neurons. All the NCX isoforms were also expressed in A-, peptidergic C-, and non-peptidergic C-neurons, and located not only in the somata, dendrites, axons and perinuclear region, but also in axons innervating the dental pulp. Reverse NCX activity was clearly observed in TG neurons. The inactivation kinetics of voltage-dependent Na^+ ^channels were prolonged by NCX inhibitors when [Ca^2+^]_i _in TG neurons was elevated beyond physiological levels.

**Conclusions:**

Our results suggest that NCXs in TG neurons play an important role in regulating Ca^2+^-homeostasis and somatosensory information processing by functionally coupling with voltage-dependent Na^+ ^channels.

## Background

Intracellular Ca^2+ ^is a primary factor in the regulation of many signal transduction pathways, and its level is precisely regulated by the Ca^2+ ^extrusion system. Active Ca^2+ ^efflux/extrusion via this system in plasma membrane involves either high-affinity, low-capacity Ca^2+^-ATPase (PMCA) or low-affinity, high-capacity Na^+^-Ca^2+ ^exchangers (NCX) [1]. The latter is a bidirectional transporter that catalyzes the electrogenic exchange of 3 Na^+ ^for 1 Ca^2+^, depending on the electrochemical gradient of the substrate ions [1-4]. These exchangers play an important role in the regulation of intracellular free Ca^2+^ concentration ([Ca^2+^]_i_) in both excitable and non-excitable cells and pump Ca^2+^ out of cells by means of the Na^+^ concentration gradient across the cell membrane [2-9]. Mammalian NCXs comprising NCX1, NCX2, and NCX3 constitute the multigene superfamily SLC8 encoded by three separate genes [1,3,10].

Somatosensory neurons of the peripheral nervous system, including dorsal root ganglion (DRG) and cranial neurons, are involved in conduction of sensory information from the peripheral organs, and relay to the central nervous system. Noxious and innocuous stimuli applied to the orofacial area are mainly received by trigeminal ganglion (TG) neurons. Both DRG and TG neurons consist of subpopulations of mechanosensitive and nociceptive neurons [11], which are divided into thickly-myelinated, fast-conducting Aβ-neurons and slower-conducting Aδ-neurons; C-neurons are unmyelinated and slow-conducting. While 90% of C-neurons and 70% of Aδ-neurons are involved in nociception, 80% of Aβ-neurons are involved in conduction of non-nociceptive information [12].

Expression of NCXs has been demonstrated in small- and large-diameter neurons of the DRG [13,14]. These NCXs usually function in Ca^2+^-efflux mode (forward mode) depending on extracellular Na^+ ^concentration, while in Ca^2+^-influx mode (reverse mode) they depend on a depolarization-induced action potential in neurons [3]. Ca^2+^-influx induces neurotransmitter release and activation of various signal transduction pathways [15]. The Ca^2+^-influx mode of NCXs has been associated with peripheral nerve injury-induced neuropathic pain [16]. Neuropathic pain is debilitating and chronic, and responds poorly to therapy. Its pathology includes a complex array of interrelated pathways leading to peripheral sensitization. A number of key factors have been hypothesized to modulate clinical status in the neuropathic pain mechanism. The NCX has been proposed as one such factor, and has been targeted in the management of peripheral nerve injury-induced neuropathic pain [16]. However, in TG neurons, PMCA has been identified as the major mechanism for removal of cytosolic Ca^2+ ^following electrical activity, and the role of NCXs in this process remains to be clarified [17]. The purpose of this study was to investigate the expression/localization of NCXs and their functional properties in TG neurons.

## Results

### Characterization of primary cultured TG neurons

**Figure 1:**
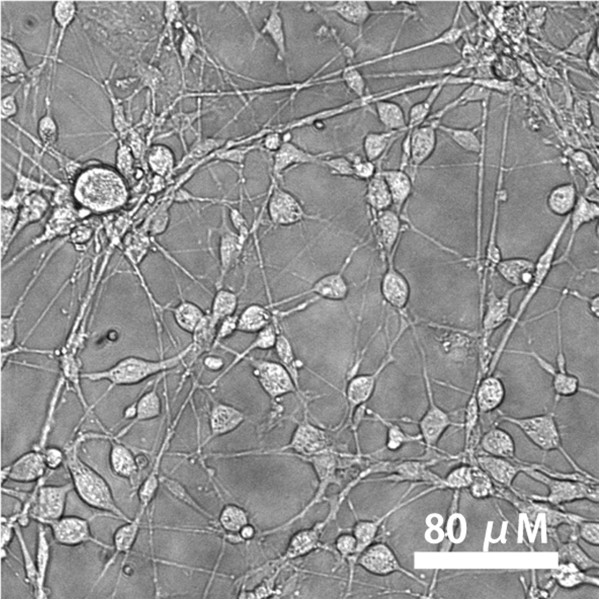
**Primary cultured TG cells.** Representative bright-field image of primary TG cultures by phase-contrast.

**Figure 2:**
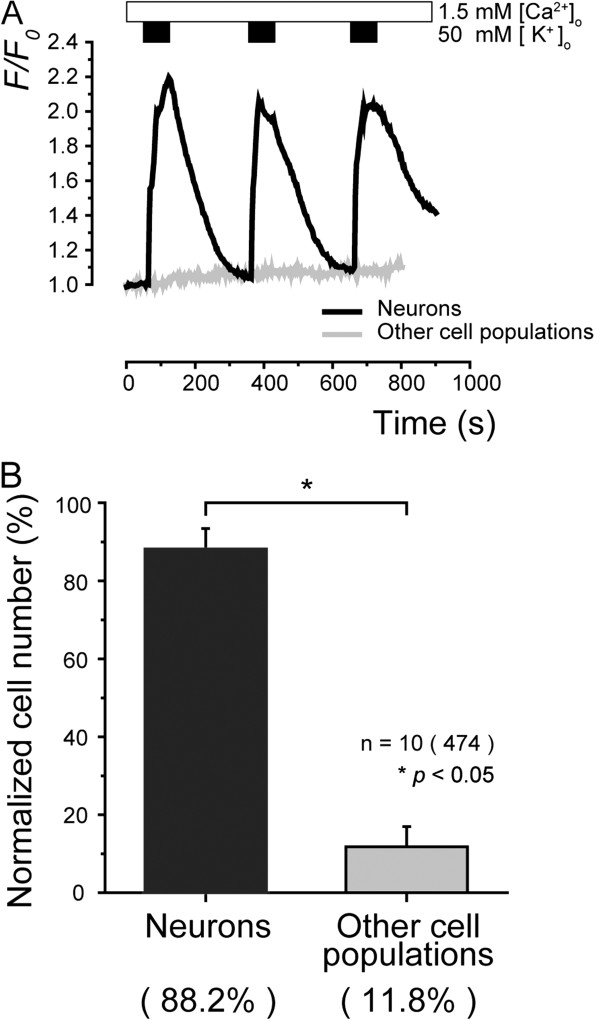
**Characterization of primary TG cultures.** (**A**) In presence of extracellular Ca^2+^ (1.5 mM; upper white box), application of 50 mM KCl solution (upper black boxes) induced an increase in [Ca^2+^]_i _in TG neurons (black solid line), while no increase in [Ca^2+^]_i _was observed in other types of cell in TG cultures (grey solid line). (**B**) Summary bar graphs showing normalized cell numbers tested. In these TG cultures, cells responding to high-KCl solution-induced depolarization with neuronal properties occupied 88.2% (black column), while other cell populations occupied 11.8% (grey column). Data points represent mean ± S.E. of number of separate experiments (numbers in parentheses represent number of tested cells). Statistically significant differences between columns are indicated by asterisk: *p<0.05.

Primary cultured TG cells (Figure 1, in phase contrast) were loaded with fura-2, and [Ca^2+^]_i _was measured. The changes in [Ca^2+^]_i _was expressed as F/F_0 _units, with R_F340/F380 _values (F) normalized with respect to the resting value (F_0_). To confirm that the primary cultured cells were indeed TG neurons, we assessed the cellular response to an extracellular solution containing high extracellular K^+^, which would induce depolarization, and hence Ca^2+ ^influx in neurons (according to the K^+^ equilibrium potential changes) [17-19]. A series of applications of high-KCl (50 mM K^+^) solutions induced an increase in [Ca^2+^]_i _in 88.2% of tested primary cultured TG cells, while the remaining cells (11.8%) showed no response (Figure 2A and B).

### mRNA expression of NCX isoforms in primary cultured TG neurons

**Figure 3:**
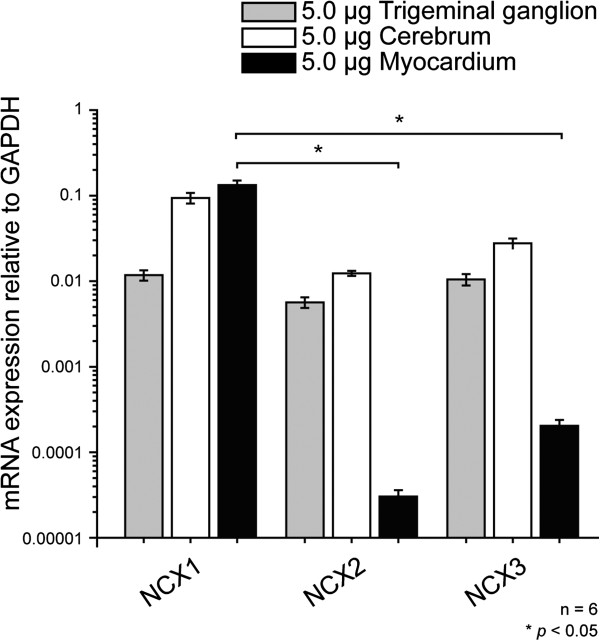
**Detection of NCX isoforms in rat TG cells by real-time RT-PCR.** PCR products were monitored by the increase in fluorescence caused by binding of SYBR green dye to double-stranded DNA. Data was analyzed by the 2^−[*ΔΔ*Ct] ^method, with GAPDH as an internal control (which was positive in all samples). Note that the relative mRNA expression level of NCX isoforms does not indicate the absolute level of expression. Each bar denotes mean ± S.D. of mRNA expression relative to GAPDH. Statistically significant differences between columns are indicated by asterisk: *p<0.05.

Gene-specific primers for real-time RT-PCR analyses revealed the expression of NCX1, NCX2, and NCX3 mRNA in rat TG neurons (gray bars in Figure 3). We did not find any significant differences in the level of mRNA expression between the 3 NCX isoforms in TG neurons, or in cells in the cerebrum. However, myocardial cells showed significantly higher expression of NCX1 mRNA compared to NCX2 and NCX3 mRNA (black bars in Figure 3).

### Immunolocalization of NCX isoforms in primary cultured TG neurons

**Figure 4:**
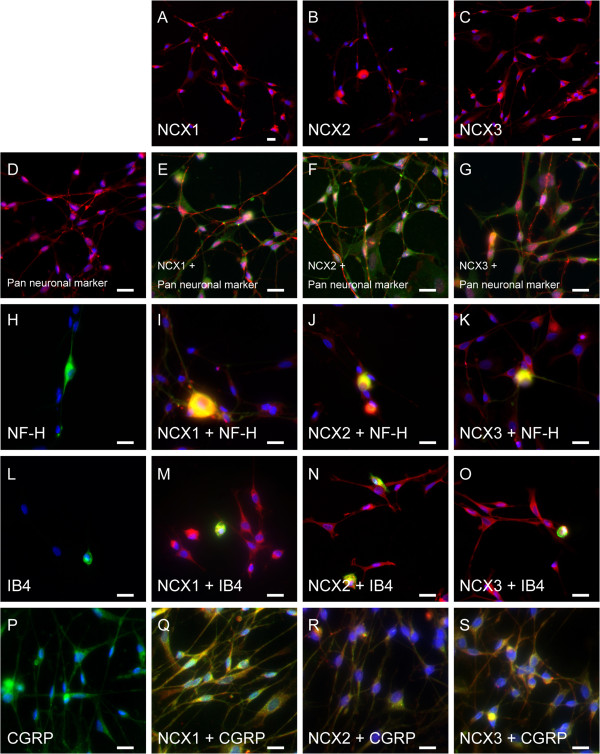
**Immunolocalization of NCX isoforms in primary cultured TG neurons.** (**A**, **B**, and **C**) NCX immunoreactivity (red) and nuclei (blue). Intense immunoreactivity was observed for NCX1 (**A**), NCX2 (**B**), and NCX3 (**C**) in TG neurons. (**D**) Positive control for Pan neuronal marker in TG neurons. Pan neuronal marker includes anti-NeuN, anti-MAP2, anti-βIIItubulin, and anti-neurofilament 200 (NF-H) antibodies. (**E**, **F**, and **G**) Double immunofluorescence staining of NCX isoforms (green) and Pan neuronal marker (red). All NCX isoforms were localized on the somata, dendrites, axons, and perinuclear region of TG neurons. (**H**) Positive immunoreactivity to NF-H as an A-neuron marker in TG neurons. (**I**, **J**, and **K**) Double staining of NCX isoforms (red) and NF-H (green). (**L**) Positive immunoreactivity to isolectin B4 (IB4; as a non-peptidergic C-neuron marker) in TG neurons. (**M**, **N**, and **O**) Double staining of NCX isoforms (red) and IB4 (green). (**P**) Positive immunoreactivity to calcitonin gene-related peptide (CGRP; as a peptidergic C-neuron marker) in TG neurons. (**Q**, **R**, and **S**) Double staining of NCX isoforms (red) and CGRP (green). Intense immunoreactivity for all of NCX isoforms was observed on A-, non-peptidergic C-, and peptidergic C-neurons. No fluorescence was detected in the negative control (data not shown). Scale bar: 20 μm.

Intense immunoreactions for NCX1, NCX2, and NCX3 were observed (red in Figure 4A to C) in the somata, dendrites, axons, and perinuclear region of primary cultured TG neurons. These cells all showed positive immunoreactivity to a blended neuronal marker cocktail containing anti-neuronal nuclei (NeuN), anti-microtubule-associated protein 2 (MAP2), βIIItubulin, and anti-neurofilament 200 (NF-H) (Figure 4D); they also showed positive immunoreactivity to either NF-H (as an A neuron marker; Figure 4H), isolectin B4 (IB4) (as a non-peptidergic C neuron marker; Figure 4L), or calcitonin gene-related peptide (CGRP) (as a peptidergic C neuron marker; Figure 4P) alone. All NCX isoforms (green in Figure 4E to G) were co-expressed with blended neuronal marker cocktails (red in Figure 4E to G). Furthermore, all NCX isoforms (red in Figure 4I to K, M to O and Q to S) were expressed on NF-H- (green in Figure 4I to K), IB4- (green in Figure 4M to O), or CGRP-positive neurons (green in Figure 4Q to S).

### Immunolocalization of NCX isoforms in the somata of TG neurons

**Figure 5:**
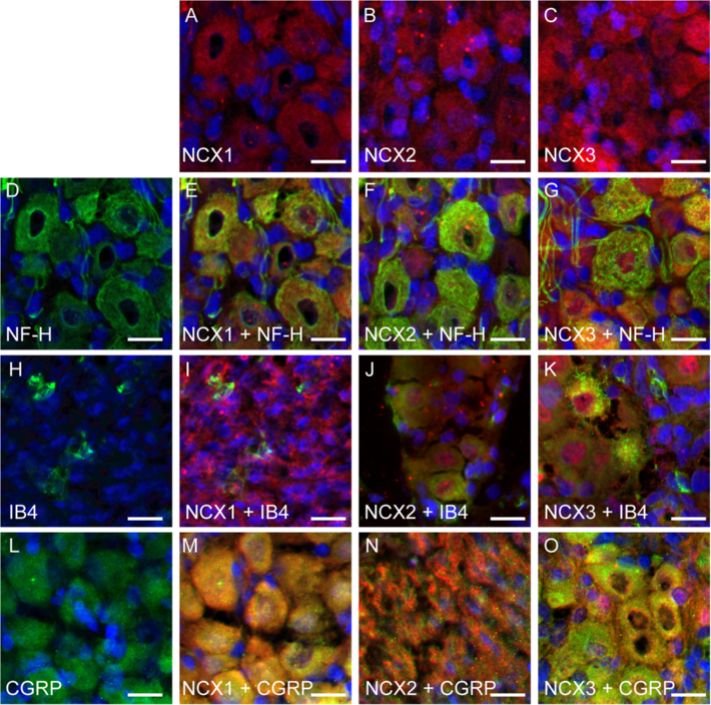
**Immunolocalization of NCX isoforms in somata of TG in cryosections.** (**A**, **B**, and **C**) NCX immunoreactivity (red) and nuclei (blue). Intense immunoreactivity was observed for NCX1 (**A**), NCX2 (**B**), and NCX3 (**C**) on entire cell bodies of TG cells. (**D**) Positive immunoreactivity to NF-H as an A-neuron marker in TG neurons. (**E**, **F**, and **G**) Double staining of NCX isoforms (red) and NF-H (green). (**H**) Positive immunoreactivity to isolectin B4 (IB4; as a non-peptidergic C-neuron marker) in TG neurons. (**I**, **J**, and **K**) Double staining of NCX isoforms (red) and IB4 (green). (**L**) Positive immunoreactivity to calcitonin gene-related peptide (CGRP; as a peptidergic C-neuron marker) in TG neurons. (**M**, **N**, and **O**) Double staining of NCX isoforms (red) and CGRP (green). NF-H-, IB4-, and CGRP-positive neurons each expressed all 3 NCX isoforms. No fluorescence was detected in the negative control (data not shown). Scale bar: 20 μm.

In cryosections, intense immunoreactivity for NCX1, NCX2, and NCX3 was observed on entire cell bodies, including the perinuclear region, of the TG cells (red in Figure 5A to C). In addition, these cells showed positive immunoreactivity for NF-H (green in Figure 5D), IB4 (green in Figure 5H), or CGRP (green in Figure 5L) alone. All the NCX isoforms (red in Figure 5E to G, I to K, and M to O) were expressed in NF-H- (green in Figure 5E to G), IB4- (green in Figure 5I to K) and CGRP-positive neurons (green in Figure 5M to O).

### Ca^2+^-dependence of reverse NCX activity

**Figure 6:**
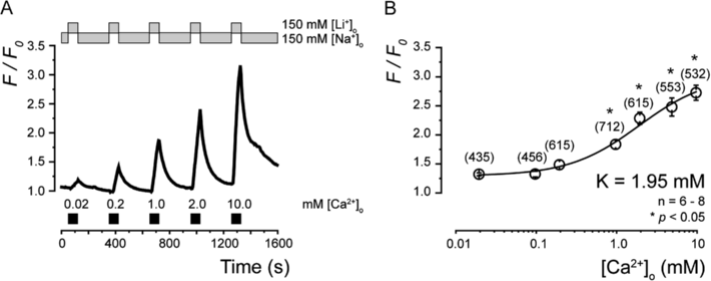
**Ca**^**2+**^**-dependence of NCX expressed in TG neurons.** (**A**) Ca^2+^ influx by NCX in TG activated by substituting Na^+^-ECS with Li^+^-ECS while maintaining osmotic strength by replacing NaCl with LiCl. Applied solution-changing protocol for [Na^+^]_o _and [Li^+^]_o_ is shown in upper grey boxes; protocol for [Ca^2+^]_o_ is shown in lower black boxes. (**B**) Concentration-response relationship for [Ca^2+^]_o_. Data points illustrate F/F_0 _values as function of applied concentration of extracellular Ca^2+^. Data points represent mean ± S.E. of number of separate experiments (numbers in parentheses represent number of tested cells). Curve (solid line) on semilogarithmic scale was fitted to *Equation1* described in text. A_max _is maximal F/F_0_ (3.02); A_min _is minimal F/F_0_ (1.30); h is 1.0. Statistically significant differences in F/F_0 _values recorded between each concentration of 0.02 mM, 0.1 mM, 0.2 mM, 1.0 mM 2.0 mM, 5.0 mM, 10 mM and 0 mM [Ca^2+^]_o_ are indicated by asterisk: *p<0.05.

In order to investigate Ca^2+ ^influx resulting from reverse NCX activity, the [Ca^2+^]_i _response was measured by equimolar substitution of Li^+ ^for Na^+^. TG neurons were first bathed in medium containing 150 mM Na^+ ^(Na^+^-ECS), and then Na^+ ^was replaced with equimolar Li^+ ^(Li^+^-ECS) in order to reverse the Na^+ ^gradient across the plasma membrane in the presence of extracellular Ca^2+ ^(Figure 6A). An increase in [Ca^2+^]_i _in the TG neurons was induced by the addition of 7 different concentrations of [Ca^2+^]_o _(0.02-10.0 mM) (Figure 6B). The increase in [Ca^2+^]_i _caused by Ca^2+ ^influx via NCX was clearly [Ca^2+^]_o_ dependent. The semi logarithmic plot (Figure 6B) illustrates F/F_0_ values as a function of applied [Ca^2+^]_o_, with an equilibrium binding constant of 1.95 mM (solid line in Figure 6B).

### Pharmacological characteristics of NCX-mediated Ca^2+ ^influx

**Figure 7:**
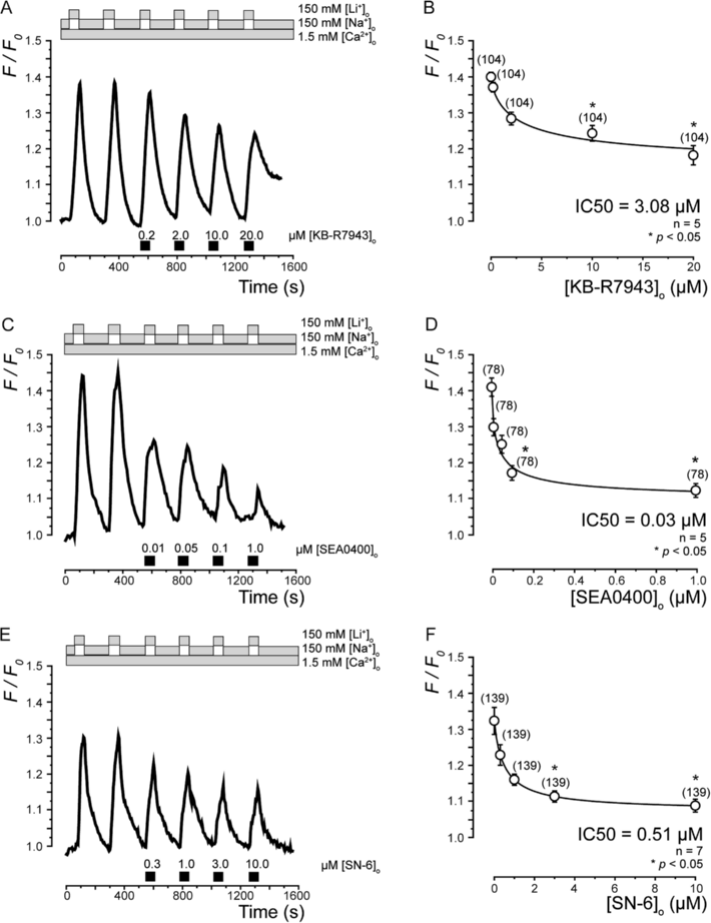
**Pharmacological identification of NCX in TG neurons.** Dose-dependent inhibition of NCX by KB-R7943 (**A**, **B**), SEA0400 (**C**, **D**), or SN-6 (**E**, **F**). (**A**, **C**, and **E**) Ca^2+^ influx by NCX was reversibly inhibited by KB-R7943 (**A**) SEA0400 (**C**), or SN-6 (**E**) in concentration-dependent manner. External solution-changing protocol of [Na^+^]_o _alone and for [Li^+^]_o _with 1.5 mM [Ca^2+^]_o _is shown in upper grey boxes in **A**, **C**, and **E**. Black bars in lower panels of each figure indicate times of application of these inhibitors to external solution. (**B**, **D**, and **F**) Dose–response relationships for inhibitory effects of KB-R7943 (**B**), SEA0400 (**D**), or SN-6 (**F**) on Ca^2+ ^influx by NCX. Data points in each figure illustrate F/F_0_ values as function of applied concentration of inhibitors and represent mean ± S.E. of number of separate experiments (numbers in parentheses represent number of tested cells). Curves (solid lines) were fitted according to *Equation1* described in text. A_max_ is maximal F/F_0_; A_min_ is minimal F/F_0_. Statistically significant differences in F/F_0_ values recorded before and after application of each concentration of inhibitors are indicated by asterisk: *p<0.05.

To determine the sensitivity of the expressed NCXs to various NCX inhibitors, we examined the effects of KB-R7943 (Figure 7A), SEA0400 (Figure 7C), and SN-6 (Figure 7E) on Ca^2+^ influx mediated by NCXs. In the presence of 1.5 mM [Ca^2+^]_o_, a series of applications of KB-R7943, SEA0400, or SN-6 significantly blocked Ca^2+ ^influx by reverse NCX activity in a concentration-dependent manner (Figure 7A, C and E). A 50% blockage of inhibitory concentration (IC_50_) was obtained at a KB-R7943 concentration of 3.08 μM (Figure 7B), an SEA0400 concentration of 0.03 μM (Figure 7D), or an SN-6 concentration of 0.51 μM (Figure 7F).

### K^+^-dependence of Na^+^-Ca^2+^ exchangers in TG neurons

**Figure 8:**
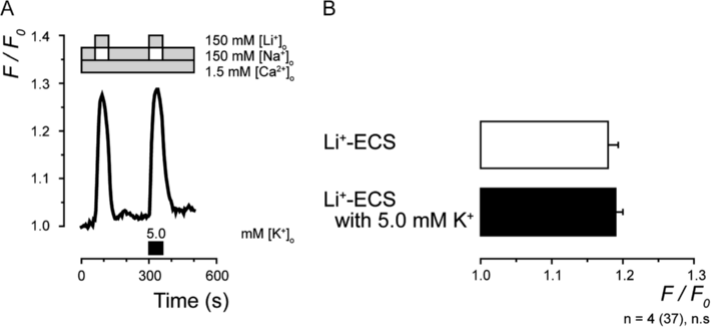
**K**^**+**^**-dependence of NCXs in TG neurons.** (**A**) Representative trace of an increase in [Ca^2+^]_i _by substituting Na^+^-ECS with Li^+^-ECS while maintaining osmotic strength by replacing NaCl with LiCl, with or without application of 5.0 mM [K^+^]_o_. Applied solution-changing protocol for [Na^+^]_o_, [Li^+^]_o_ and [Ca^2+^]_o_ is shown in upper grey boxes; protocol for [K^+^]_o_ is shown in lower black boxes. (**B**) Summary bar graphs of increase in [Ca^2+^]_i _by application of Li^+^-ECS without (open column) or with (black column) 5.0 mM extracellular K^+^. Each bar denotes mean ± S.E. of number of separate experiments (numbers in parentheses represent number of tested cells). No statistically significant difference in F/F_0 _values was observed between Li^+^-ECS without or with 5.0 mM K^+^.

In order to investigate K^+^-dependence of Ca^2+ ^influx mediated by reverse Na^+^-Ca^2+^ exchange activity, the [Ca^2+^]_i_ response was measured by equimolar substitution of Li^+ ^for Na^+^ with or without extracellular 5.0 mM K^+^. In the presence of 1.5 mM [Ca^2+^]_o_, application of Li^+^-ECS either with or without extracellular 5.0 mM K^+^ induced a robust increase in [Ca^2+^]_i_ in the TG neurons (Figure 8A). No significant differences were observed in these [Ca^2+^]_i_ responses (Figure 8B).

### Prolonged inactivation kinetics in voltage-dependent Na^+ ^channels induced by NCX blockage

**Figure 9:**
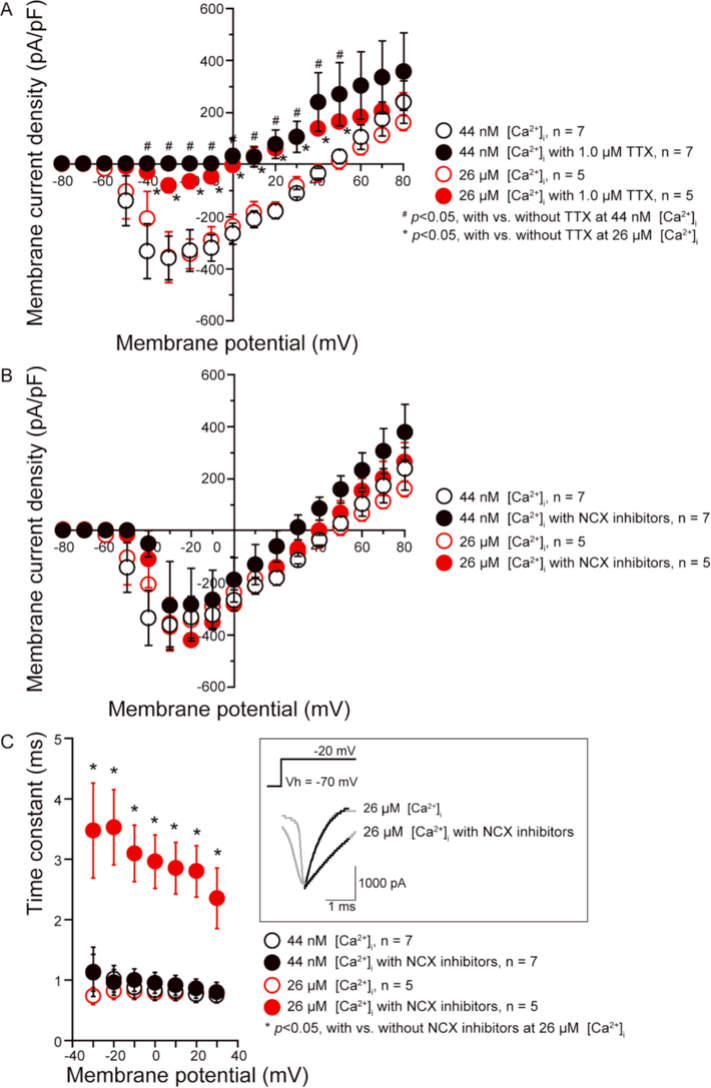
**Prolonged inactivation kinetics in voltage-dependent Na**^**+ **^**channels caused by NCX blockage.** (**A** and **B**) I-V relationships were obtained by plotting the values of peak current against applied membrane potentials, which cell size accounted by normalizing for measured capacitance and expressing current amplitudes in terms of current densities (pA/pF). Cell size was 22–31 μm. V_h_ was −70 mV. (**A**) Inward currents (open circles) were inhibited by 1.0 μM TTX (filled circles) under both the physiological condition with 44 nM [Ca^2+^]_i_ (black circles), and the [Ca^2+^]_i_-overloaded condition with 26 μM [Ca^2+^]_i_ (red circles), indicating that currents were voltage-dependent Na^+^ currents (total *I*_Na_). (**B**) A cocktail of NCX inhibitors (3.0 μM KB-R7943, 0.03 μM SEA0400, and 0.5 μM SN-6) (filled circles) had no significant effect on the peak amplitude of total *I*_Na _in either the physiological (black circles) or [Ca^2+^]_i_-overloaded (red circles) condition. (**C**) Comparison of the time constant of inactivation (τ) for total *I*_Na _with (filled circles) or without (open circles) the cocktail of NCX inhibitors under the physiological (black circles) and [Ca^2+^]_i_-overloaded (red circles) conditions, at membrane potentials ranging from −30 mV to +30 mV. (Inset in **C**) Total *I*_Na _in the [Ca^2+^]_i_-overloaded condition (grey lines), evoked by a voltage pulse of up to −20 mV from a V_h _of −70 mV, was well fitted by a single exponential function (black line). An inward current at −20 mV showed rapid inactivation without the cocktail of NCX inhibitors, while the NCX inhibitors slowed the inactivation kinetics. Data points (**C**) illustrate τ as a function of each membrane potential. Data points (**A** to **C**) represent mean ± S.D. of the number of tested cells. Statistically significant differences in current amplitude values or τ of total *I*_Na_ inactivation are indicated.

The effect of NCX inhibitors on voltage-dependent Na^+ ^currents (*I*_Na_) was examined to investigate the contribution of NCX activity to excitability in TG neurons. Inward currents were elicited from TG neurons in 20-ms voltage clamp steps ranging from −80 to +80 mV. Voltage steps were applied in 10-mV increments from a holding potential (V_h_) of −70 mV, in the presence of standard extracellular solution, and intracellular solution (ICS) containing either 44 nM, or 26 μM free Ca^2+^ (physiological or [Ca^2+^]_i_-overloaded condition, respectively). Current–voltage (I-V) relationships were determined by plotting the peak current amplitudes against the applied membrane potentials (circles; Figure 9A and B). Differences in cell size were accounted for by normalizing for the measured capacitance, and current amplitudes were expressed in terms of current densities (pA/pF). At a V_h_ of −70 mV, inward currents developed at a membrane potential of −60 mV and reached maximum amplitude at a membrane potential of −30 mV under both conditions (44 nM and 26 μM [Ca^2+^]_i_, open black and red circles; Figure 9A and B). There was no significant difference between the current densities under the physiological (44 nM [Ca^2+^]_i_) and [Ca^2+^]_i_-overloaded conditions (26 μM [Ca^2+^]_i_). Inward currents were, however, significantly blocked by the voltage-dependent Na^+^ channel blocker tetrodotoxin (TTX) (1.0 μM) under both conditions (filled black and red circles; Figure 9A), while a small residual TTX-resistant component was observed. This indicates that these currents (total *I*_Na_) were carried mainly by TTX-sensitive voltage-dependent Na^+ ^channels, with small component carried by TTX-resistant channels.

As shown in Figure 9B, in the physiological condition (open and filled black circles), there was no significant change in the peak amplitude of total *I*_Na _without (open black) or with (filled black) a cocktail of NCX inhibitors (3.0 μM KB-R7943, 0.03 μM SEA0400 and 0.5 μM SN-6). This was also the case in the [Ca^2+^]_i_-overloaded condition (open and filled red circles in Figure 9B). The mean peak current density of total *I*_Na_ at a membrane potential of −30 mV in the physiological condition was −363.3 ± 84.2 pA/pF or −355.0 ± 98.0 pA/pF in the absence or presence of the cocktail of NCX inhibitors, respectively. Those in the [Ca^2+^]_i_-overloaded condition were −289.9 ± 170.2 pA/pF in the absence of or −368.0 ± 93.4 pA/pF in the presence of the cocktail.

To further investigate the effect of NCX inhibitors on total *I*_Na_, the inactivation kinetics were examined by analyzing current decay during depolarization. The time-course of inactivation with or without the cocktail of NCX inhibitors was well described by the single exponential function. In the absence of NCX inhibitors, we did not observe any significant changes in the mean values for the time constants of inactivation (τ) of total *I*_Na _between the physiological (open black circles) and [Ca^2+^]_i_-overloaded condition (open red circles), as shown in Figure 9C. In the presence of NCX inhibitors, there were no significant changes in the values of τ in the physiological condition (filled black circles), compared with those in the absence of NCX inhibitors (open black circles in Figure 9C). However, as shown in the inset of Figure 9C, under the [Ca^2+^]_i_-overloaded condition and in the presence of NCX inhibitors, the values of τ (black lines as described by the single exponential function) were significantly prolonged in a voltage-dependent manner (filled red circles in Figure 9C).

### Immunolocalization of NCX isoforms in axons innervating dental pulp

**Figure 10:**
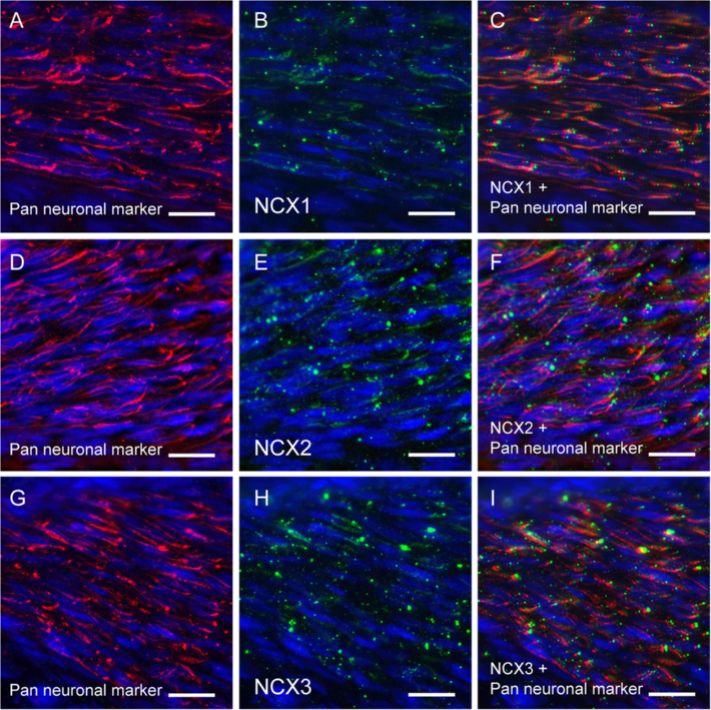
**Immunolocalization of NCX isoforms in axons innervating dental pulp.** (**A**, **D**, and **G**) Pan neuronal marker immunoreactivity (red) and nuclei (blue). Intense immunoreactivity was observed for Pan neuronal marker in axons innervating the dental pulp. Pan neuronal marker includes anti-NeuN, anti-MAP2, anti-βIIItubulin, and anti-neurofilament 200 (NF-H) antibodies. (**B**, **E**, and **H**) Immunoreactivity for NCX1 (**B**), NCX2 (**E**), and NCX3 (**H**). (**C**, **F**, and **I**) Double staining of Pan neuronal marker (red) and NCX isoforms (green). All NCX isoforms were expressed on Pan neuronal marker-positive neurons. No fluorescence was detected in the negative control (data not shown). Scale bar: 20 μm.

We further investigated the distribution of NCX isoforms in the axons of TG neurons innervating peripheral tissue. Intense immunoreactivity for the Pan neuronal maker was observed on axons innervating dental pulp (red in Figure 10A, D, and G), and this was co-localized with positive immunoreactivity for NCX1 (green in Figure 10B and C), NCX2 (green in Figure 10E and F), and NCX3 (green in Figure 10H and I).

## Discussion

Our results demonstrate the functional expression of NCX1, NCX2, and NCX3 in TG neurons. Application of a high-KCl solution resulted in a depolarization-induced increase in [Ca^2+^]_i _in 90% of the primary cultured TG cells. These depolarization-induced increases in [Ca^2+^]_i _were observed in all tested cells showing reverse NCX activity (data not shown). In addition, these cells, which were obtained from rat TG primary cultures, expressed total *I*_Na_, indicating that the tested cells comprised mostly TG neurons rather than glial cells [17-19].

Real-time RT-PCR revealed mRNA expression of all NCX isoforms in the primary cultured TG neurons. NCX1 was ubiquitously characterized and cloned (in organs such as the heart, brain, and kidney and at lower levels in other tissues), while NCX2 and NCX3 were selectively expressed in the brain, skeletal muscle, and selected neuronal populations [1,3,4,10]. In the current study, NCX1, NCX2, and NCX3 expression pattern in the TG neurons was similar to that in the cerebrum [3], but not to that in myocardium. Expression of all NCX isoforms has been reported to be much higher in neurons than in astrocytes [20,21].

Intense NCX immunoreactivity was observed on the somata, dendrites, axons and perinuclear region of TG neurons (Figures 4 and 5). These NCX isoforms appeared to be localized on the cell membrane rather than in the cytoplasm. However, since mitochondrial expression of NCXs has been demonstrated [22], we could not exclude the possibility that NCX isoforms were expressed and localized on the intracellular organelles, including mitochondria. In addition, NF-H-positive A neurons, IB4-positive non-peptidergic C neurons, and CGRP-positive peptidergic C neurons each expressed all of 3 NCX isoforms (Figure 5). Moreover, expression of NCXs was observed on axons innervating the peripheral tissue (dental pulp), indicating that all NCX isoforms are distributed throughout trigeminal A-, non-peptidergic C-, and peptidergic C-neurons [12].

It has been reported that NF-H (a marker for myelinated medium- to large-diameter afferents) [23] and IB4 (which binds to unmyelinated small-diameter nociceptive afferent neurons) [24,25] co-localize with P2X_2 _and P2X_3 _receptors in rat TG neurons, as Aδ and C nociceptors, respectively [26,27]. In addition, CGRP is released from primary afferent terminals onto secondary afferents in response to various noxious stimulation [24,28]. Considering these findings, the current results indicate that NCX isoforms are expressed not only in mechanosensitive Aβ neurons, but also in nociceptive afferent neurons.

Pharmacological identification of NCX was achieved by using benzyloxyphenyl derivatives, which are NCX inhibitors. The potencies of 3 benzyloxyphenyl derivatives, KB-R7943, SEA0400, and SN-6, on Na^+^-dependent Ca^2+ ^uptake have been reported in fibroblasts [29]. These 3 inhibitors partially block Ca^2+^ influx through NCX and have different isoform selectivities. KB-R7943 is 3-times more potent against NCX3 than against NCX1 or NCX2, while SEA0400 predominantly inhibits NCX1, only mildly inhibits NCX2, and exerts almost no influence upon NCX3 [29]. SN-6 shows 3- to 5-times greater inhibition of NCX1 than either NCX2 or NCX3 [29]. In the present study, the values of IC_50 _for KB-R7943 (3.08 μM) and SEA0400 (0.056 μM) were consistent with the general characteristics of those reported for fibroblasts transfected with NCX1, NCX2, or NCX3. However, reverse NCX activity in TG neurons showed a strong sensitivity to SN-6 (IC_50_=0.51 μM). Therefore, our results suggest that NCX1 is the predominantly functional isoform in TG neurons, although in TG neurons, the mRNA expression levels of the 3 isoforms were not different. During postnatal development, however, the expression pattern of NCX isoforms in rat cultured neurons changes: NCX1 was predominant in neonatal rat cortical cultured neurons, but the level of NCX2 increased, while NCX1 and NCX3 level decreased in adult rat neurons [21]. These results are in line with our results obtained from neonatal (4–7 days old) rat TG neurons. Further study is required to precisely characterize the developmental changes in NCX isoform expressions in TG neurons.

In addition to NCX, the intracellular Ca^2+^ efflux system on the plasma membrane involves another type of transporter, the K^+^-dependent Na^+^-Ca^2+ ^exchangers (NCKXs) which are comprises NCKX1 to NCKX5 and constitutes the SLC24 gene family [30,31]. The NCKXs are also reportedly distributed throughout the central nervous system [32-34]; however, Ca^2+ ^influx by reverse Na^+^-Ca^2+ ^exchange did not show any K^+^-dependence (Figure 8), indicating that NCKX activities did not contribute to the reverse NCX activities recorded in the present study.

We also investigated the coupling mechanism between voltage-dependent Na^+ ^channels and NCXs in TG neurons. In the nociceptive neurons, voltage-dependent Na^+ ^channels are pharmacologically categorized as either TTX-sensitive or TTX-resistant, depending on their activation and inactivation kinetics, activation threshold, and voltage dependence of activation and steady-state inactivation [35-37]. In this study, although NCX expression in small- to large-diameter neurons was observed to co-localize with NF-H-, IB4- and CGRP-positive TG neurons, voltage-dependent inward currents were significantly blocked by TTX. This indicates that the inward currents were carried primarily by TTX-sensitive voltage-dependent Na^+ ^channels; however, we could not separate total *I*_Na _into TTX-sensitive and TTX-resistant components.

As an essential pain modulator, NCXs are of interest in the management of peripheral nerve injury-induced neuropathic pain [16]. NCX activation in Ca^2+^-influx mode is associated with [Ca^2+^]_i_ increase and various pathological conditions, including hypoxia-anoxia, white matter degeneration after spinal cord injury, brain trauma, and optical nerve injury [38]. An overload of [Ca^2+^]_i_ via NCXs in Ca^2+^-influx mode driven by axonal Na^+^ accumulation via TTX-sensitive voltage-dependent Na^+^ channels has been observed in injured myelinated axons in dorsal root [39]. This increase in [Ca^2+^]_i _was not dependent on L-type voltage-dependent Ca^2+^ channels [39]. In addition, in the case of axonal injury, stretch-injured traumatic deformation of axons induces abnormal Na^+ ^influx via TTX-sensitive voltage-dependent Na^+^ channels, which subsequently triggers an increase in intra-axonal Ca^2+ ^via the activation of NCX in Ca^2+^-influx mode [40]. In an anoxic situation, myelinated axons lose K^+^, while intra-axonal Na^+^ concentrations increase primarily via Na^+^ influx through TTX-sensitive voltage-dependent Na^+^ channels; elevated axoplasmic Na^+^ and axolemmal depolarization promote Ca^2+^ influx mediated primarily by NCX reverse activity [41]. This pathway is considered the primary mechanism of Ca^2+^ influx in neuropathic pain states [41].

In our study, the [Ca^2+^]_i_-overloaded condition had no effect on either peak current amplitudes, or inactivation kinetics of total *I*_Na _(black and red open circles in Figure 9A and C). Additionally, in the physiological condition, the inhibition of NCXs did not influence the peak amplitudes or inactivation kinetics of total *I*_Na _(see Figure 9B and C). These findings indicate that neither [Ca^2+^]_i_-overload itself nor NCX inhibition seem to be directly related to neuronal excitability; rather our results support previous findings (see above) [41] that [Ca^2+^]_i_-overload caused by Ca^2+^ influx via NCXs contributes to neuropathic pain. In contrast, the inactivation process of *I*_Na _was significantly and voltage-dependently prolonged by the application of NCX inhibitors, only when the [Ca^2+^]_i_ in TG neurons was overloaded (red filled circles in Figure 9C), suggesting that NCXs play important roles in maintaining the normal functioning of voltage-dependent Na^+^ channels in TG neurons under the pathologically [Ca^2+^]_i_-overloaded condition, but not under physiological conditions. Therefore, not only [Ca^2+^]_i_-overload from Ca^2+^ influx via NCXs, but also the reduced activity of NCXs under pathological [Ca^2+^]_i_-overload in TG neurons might be involved in modulating neural excitability.

In contrast, KB-R7943 (at 10 μM), but not SEA0400, has been shown to have an inhibitory effect on the peak amplitude of *I*_Na _in myocardium [42]. In our study, at the IC_50_ for inhibition of the reverse Ca^2+^ influx mode of NCX, not only KB-R7943, but also SEA0400 and SN-6 did not significantly affect the *I*_Na _peak current density in TG neurons, under both the physiological and [Ca^2+^]_i_-overloaded conditions. In addition, a cocktail of these 3 NCX inhibitors (KB-R7943, SEA0400 and SN-6) prolonged the inactivation process of *I*_Na _under the [Ca^2+^]_i_-overloaded condition, but not under the physiological condition (red filled circles in Figure 9C). Thus, a non-specific effect of these NCX inhibitors on total *I*_Na _is unlikely.

Voltage-dependent Na^+ ^channels comprise a transmembrane glycoprotein consisting of a pore-forming α subunit, and an auxiliary β subunit which modulates the kinetics and voltage dependence of Na^+ ^channel activation and inactivation [43,44]. The β subunit, in particular, regulates the propagation of action potentials through critical intermolecular and cell-cell communication events [44]. Additionally, the β subunit appears with the α subunit during early neural development, and its expression is increased during rapid neuronal growth and differentiation [45]. In DRG neurons, similar expression patterns of voltage-dependent Na^+^ channel α subunits (e.g. Na_v_1.6, Na_v_1.7, Na_v_1.8, and Na_v_1.9) and NCX2 have been demonstrated along the neurites and at the neurite tips [13]. Furthermore, NCX1 co-localizes with Na_v_1.6 in the spinal cord [46]. Therefore, the NCXs in TG neurons functionally couple with voltage-dependent Na^+^ channels, most likely via their β subunit. The current results together with those of earlier reports suggest that functional coupling between NCXs and voltage-dependent Na^+^ channels in TG neurons is one of the key factor(s) in the development and/or modulation of neuro-pathological conditions such as neuropathic pain.

## Conclusion

In the present study, we showed that TG neurons functionally expressed all NCX isoforms in axon, dendrites and somata, and that they localized on A-, non-peptidergic C-, and peptidergic C-neurons. The present results suggest that coupling of NCXs with voltage-dependent Na^+^ channels in TG neurons may play a key role in the modulation of nociceptive and/or neuropathic pain in the oral and maxillofacial region.

## Methods

### Cell isolation and primary culture

All animals were treated in accordance with the Guiding Principles for the Care and Use of Animals in the Field of Physiological Sciences approved by the Council of the Physiological Society of Japan and the American Physiological Society, and the guidelines established by the National Institutes of Health (USA) regarding the care and use of animals for experimental procedures. The study was approved by the Ethics Committee of Tokyo Dental College (approval No.232507/No.242502). The Trigeminal ganglion (TG) was dissected and isolated from neonatal Wistar rats of both sexes (4–7 days old) under pentobarbital sodium anesthesia (50 mg/kg intraperitonially). The cells were dissociated by enzymatic treatment with Hank’s balanced salt solution (Invitrogen, Grand Island, NY, USA) containing 20 U/ml papain (Worthington, Lakewood, NJ, USA) for 20 min at 37°C followed by dissociation by trituration [47,48]. Dissociated TG cells were plated onto poly-L-lysine-coated dishes (Corning, Corning, NY, USA). Primary culture was performed with Leibovitz’s L-15 medium (Invitrogen) containing 10% fetal bovine serum, 1% penicillin-streptomycin (Invitrogen), 1% fungizone (Invitrogen), 26 mM NaHCO_3 _and 30 mM glucose (pH 7.4) [47,48]. The cells were incubated and maintained at 37°C in a humidified atmosphere of 95% air and 5% CO_2 _for 24–48 hr.

### Real-time reverse transcription-polymerase chain reaction

The cerebrum and myocardium were dissected from neonatal Wistar rats of both sexes (4–7 days old) under pentobarbital sodium anesthesia. Total mRNA was extracted from the cultured rat TG cells or isolated cerebrum and myocardium by a modified acid guanidium-phenol-chloroform method [49]. Reverse transcription, cDNA amplification and polymerase chain reaction (PCR) were performed with the One-Step SYBR Primescript RT-PCR Kit and Thermal Cycle Dice for semiquantitative real-time RT-PCR (Takara-Bio, Shiga, Japan). The forward and reverse primer sets used are listed in Table 1[9]. Results obtained from real-time RT-PCR experiments were quantified using the comparative threshold (2^-[*ΔΔ*Ct]^) method (Ct represents cycle threshold) [50,51]. Relative mRNA expression level was normalized by that of glyceraldehyde-3-phosphate dehydrogenase (GAPDH). The Ct for each primer set was obtained from samples and averaged. Delta Ct represented the calculated difference between the average Ct for the target gene (NCX isoforms) and the average Ct for GAPDH as the control for total starting RNA quantity. The delta-delta Ct method of calculation was then used to assess fold-change in gene expression relative to the GAPDH gene [50,51].

**Table 1:** Primer sets used for detection of NCX in real-time RT-PCR analyses

**Sequence**	**Product size**
For real-time RT-PCR	
NCX1	109 bp
F: 5′-GTGTTTGTCGCTCTTGGAACCTC-3′	
R: 5′-CGTTGCTTCCGGTGACATTG-3′	
NCX2	184 bp
F: 5′-CAGGTCAAGATAGTGGACGACGAA-3′	
R: 5′-GAACTGGCTTGCCCATCTCTG-3′	
NCX3	92 bp
F: 5′-AGCTGTCACGGTGTGAGAATGAG-3′	
R: 5′-TGACCACAATGATGACCAGATGAA-3′	
GAPDH	143 bp
F: 5′-GGCACAGTCAAGGCTGAGAATG-3′	
R: 5′-ATGGTGGTGAAGACGCCAGTA-3′	

Conditions for real-time RT-PCR: 1 cycle of 42°C for 5 min, followed by 1 cycle of 95°C for 10 s, 40 cycles of 95°C for 5 s, and 60°C for 30 s; and for dissociation curve analysis: 1 cycle of 95°C for 15 s, 1 cycle of 60°C for 30 s, and 1 cycle of 95°C for 15 s. F: forward primer, R: reverse primer.

### Immunofluorescence analyses

In order to identify the localization of NCX isoforms in TG neurons, we examined immunofluorescence in the primary cultured TG cells, as well as in cryosections from the TG and mandible. The TG and mandible was dissected bilaterally from neonatal Wistar rats of both sexes (4–7 days old) under pentobarbital sodium anesthesia. Dissected TG and mandible were embedded together in mounting medium (Tissue-Tek O.C.T.; Sakura Finetek, Tokyo, Japan), and sectioned longitudinally on a cryostat at 15-μm thickness. Primary cultured TG cells were seeded and cultured on poly-D-lysine-coated glass slides (Matsunami, Osaka, Japan). After fixation with 50% ethanol and 50% acetone and blocking in 10% donkey serum, each section and primary cultured TG cell was incubated with goat anti-NCX1, anti-NCX2, or anti-NCX3 antibodies (Santa Cruz Biotechnology, Santa Cruz, CA, USA; dilution, 1:50) and a blended neuronal maker cocktail (Milli-Mark™ Pan Neuronal Marker, Millipore, Billerica, MA, USA; dilution 1:50) including mouse anti-NeuN, anti-MAP2, anti-βIIItubulin and anti-NF-H antibodies. The TG cells were also incubated with either rabbit anti-NF-H antibody (Millipore; dilution 1:200) as an A-neuron marker, IB4 antibody-conjugated FITC (dilution 1:200) as a non-peptidergic C-neuron marker, or rabbit anti-CGRP antibody (Santa Cruz Biotechnology; dilution 1:50) as a peptidergic C-neuron marker, at 4°C overnight. The cells were then washed and incubated with a secondary antibody (Alexa Fluor 488 or 568 donkey anti-goat IgG, Alexa Fluor 568 donkey anti-mouse IgG, or Alexa Fluor 488 donkey anti-rabbit IgG, Molecular Probes, Eugene, OR, USA; dilution 1:100) for fluorescence staining and with 4’,6-diamino-2-phenylindole dihydrochloride (Molecular Probes) for nuclear staining at room temperature for 5 min. Negative controls were prepared by using nonimmune IgGs diluted at a concentration equivalent to that of the primary antibodies. Cells were examined under a conventional fluorescence microscope (Zeiss, Jena, Germany).

### Measurement of intracellular Ca^2+ ^concentration

Primary cultured TG cells were incubated for 60 min at 37°C in Hank’s solution containing 10 μM fura-2 acetoxymethyl ester (Dojindo, Kumamoto, Japan) and 0.1% (w/v) pluronic acid F-127 (Invitrogen), followed by rinsing with fresh Hank’s solution. A dish with fura-2-loaded TG neurons was mounted on the stage of a microscope (Olympus, Tokyo, Japan) incorporated into the Aquacosmos system and software (Hamamatsu Photonics, Shizuoka, Japan) equipped with an excitation wavelength selector and an intensified charge-coupled device camera (Hamamatsu Photonics). [Ca^2+^]_i _was expressed as the fluorescence ratio (R_F340/F380_) at two excitation wavelengths of 380 nm and 340 nm.

### Whole-cell recording techniques

Whole-cell patch-clamp recordings were carried out under voltage-clamp conditions [52]. Patch pipettes with a resistance of 2–5 MΩ were pulled from capillary tubes by using a DMZ-Universal Puller (Zeitz-Instruments, Martinsried, Germany) and filled with an intracellular solution (ICS). Whole-cell currents were measured by using a patch-clamp amplifier (L/M-EPC-7+; Heka Elektronik, Lambrecht, Germany). Current traces were displayed and stored in a computer by using pCLAMP (Axon Instruments, Foster City, CA) after digitizing the analog signals at 10 kHz (DigiData 1440A; Axon Instru Instruments). Current records were filtered at 3 kHz. Data were analyzed offline by using pCLAMP. The currents via voltage-dependent Na^+ ^channels (total *I*_Na_) were recorded with a holding potential of −70 mV. The current–voltage (I-V) relationship of *I*_Na _was measured by applying 20-ms depolarizing pulses increasing from −80 to +80 mV in 10-mV steps at 2-s intervals.

### Solutions and reagents

Standard recording solution comprised Hank’s balanced salt solution containing the following: 137 mM NaCl, 5.0 mM KCl, 1.5 mM CaCl_2_, 0.5 mM MgCl_2_, 0.44 mM KH_2_PO_4_, 4.17 mM NaHCO_3_, 0.34 mM Na_2_HPO_4_, and 5.55 mM glucose (pH 7.4). High-KCl solution consisting of the following was used to activate depolarization-induced [Ca^2+^]_i_ increase in TG neurons [17-19]: 91 mM NaCl, 50 mM KCl, 2.5 mM CaCl_2, _0.5 mM MgCl_2, _10 mM HEPES, 10 mM glucose, and 12 mM NaHCO_3 _(pH 7.4). Extracellular solution (Na^+^-ECS) consisted of the following: 150 mM NaCl, 0.02-10 mM CaCl_2_, 10 mM sucrose, 20 mM HEPES, (pH 7.4). To activate the Ca^2+ ^influx mode of NCX, extracellular Na^+ ^concentration ([Na^+^]_o_) in the Na^+^-ECS was substituted with equimolar extracellular Li^+^ concentration ([Li^+^]_o_) (Li^+^-ECS). To examine the K^+^-dependence of Na^+^-Ca^2+ ^exchange in TG neurons, 5.0 mM K^+^ was added to Li^+^-ECS.

The intracellular solution (ICS) for patch-clamp recordings in the physiological condition contained the following: 140 mM KCl, 2.0 mM Mg-ATP, 10 mM TEA-Cl, 1.0 mM CaCl_2_, 5.0 mM EGTA (free [Ca^2+^] was 44 nM), and 10 mM HEPES (pH 7.2). For the [Ca^2+^]_i_-overloaded condition, the intracellular solution comprised following; 130 mM KCl, 2.0 mM Mg-ATP, 10 mM TEA-Cl, 10 mM CaCl_2_, 10 mM EGTA (free [Ca^2+^] was 26 μM) [53], and 20 mM HEPES (pH 7.2). Standard solution was also used as an ECS for the patch-clamp recordings. KB-R7943 and SN-6 were obtained from Tocris Bioscience (Bristol, UK). SEA0400 (2-[4-[(2,5-difluorophenyl)methoxy]phenoxy]-5-ethoxyaniline) was synthesized by Taisho Pharmaceutical Co. Ltd (Saitama, Japan), and was a gift from Professors Toshio Matsuda and Akemichi Baba, Osaka University, Japan. These reagents were diluted in the ECSs for [Ca^2+^]_i _measurement and patch-clamp recording, and applied at the appropriate concentration to primary cultured TG neurons via a gravity-fed perfusion system (Warner instruments, Hamden, CT, USA). For Na^+ ^inward current recording, 1.0 μM tetrodotoxin was diluted with standard solution. Except for those noted above, all other reagents were obtained from Sigma-Aldrich (St. Louis, MO, U.S.A).

### Statistics and offline analysis

The Ca^2+^-dependence of NCX activity and the dose–response relationship for the inhibitory effect of NCX inhibitors were obtained by fitting the data with following function:

$${A=A}_{\max}/\left[1+\left(K\;\text{or}\;{\mathrm{IC}}_{50}/{\left[X\right]}_{o}{}^{h}\right)\right]{+A}_{\text{min}}$$

where K is the equilibrium binding constant for applied extracellular Ca^2+^ concentration ([Ca^2+^]_o_) with a Hill coefficient (h) equal to 1; or where IC_50_ is the 50% inhibitory concentration for applied NCX inhibitors. A_max _is maximal, and A_min _is minimal response. The [X]_o _indicates applied concentration of extracellular Ca^2+ ^or inhibitors. The kinetics of inactivation was determined by fitting the experimental data with a single exponential function using the pCLAMP software.

Data were expressed as the mean ± standard error (S.E.) or standard deviation (S.D.) of the mean of *n* observations, where *n* represents the number of separate experiments. The Wilcoxon t-test, Friedman test, or Kruskal-Wallis test and Dunn’s post hoc test were used to determine non-parametric statistical significance. A *p* value of less than 0.05 was considered significant. The statistical analysis was performed using Graph Pad Prism 5.0 (Graph Pad Software, La Jolla, CA, USA).

## Competing interests

The authors declare no conflict of interest regarding the subject or materials discussed in this manuscript. In addition, the funders had no role in study design, data collection, analysis, decision to publish, or preparation of the manuscript.

## Authors’ contributions

MZ, TI and YS were responsible for the conception and design of the experiments. HK, MS, US, MT, and YS were responsible for the acquisition, analysis and interpretation of the data. HK and YS were responsible for drafting and critically revising the article in terms of intellectual content. YS was responsible for final approval of the version to be submitted/published. All of the authors were involved in critically revising important intellectual content and giving final approval of the version of the manuscript to be published.
